# Ancient Relatives of Modern Maize From the Center of Maize Domestication and Diversification Host Endophytic Bacteria That Confer Tolerance to Nitrogen Starvation

**DOI:** 10.3389/fpls.2021.660673

**Published:** 2021-09-16

**Authors:** Christopher R. Dumigan, Jade Muileboom, Jake Gregory, Anuja Shrestha, Omar A. Hewedy, Manish N. Raizada

**Affiliations:** Department of Plant Agriculture, University of Guelph, Guelph, ON, Canada

**Keywords:** endophyte, diazotroph, nitrogen, microbiome, maize, teosinte, landrace, domestication

## Abstract

Plants can adapt to their surroundings by hosting beneficial bacteria that confer a selective advantage in stressful conditions. Endophytes are a class of beneficial bacteria that exist within the internal spaces of plants and many species can improve plant nitrogen use efficiency. Nitrogen is an essential plant macronutrient, and is often a limiting factor to plant growth, especially in cereal crops such as maize. Every year farmers apply over 100 million metric tonnes of synthetic nitrogen fertilizer to meet the growing demand for stable food crops. Breeding efforts in maize over the past several decades has focused heavily on yield in response to nitrogen inputs, and so may have selected against adaptations that allow plants to survive in nitrogen stressed conditions. Data suggests that our heavy dependence on synthetic nitrogen fertilizer is not sustainable in the long term, and so there is on-going research efforts to reduce and replace this currently essential part of modern agriculture. Bacteria that improve plant tolerance to nitrogen stressed environments would allow farmers to reduce the amount of fertilizer they apply. The selection of maize under high nitrogen conditions to create modern varieties may have caused the plant to lose these beneficial bacteria that allowed wild maize ancestors to thrive in low nitrogen soil. Here in this study, we examine the root and shoot microbiomes of the wild ancestor of all maize, Parviglumis, and an ancient Mexican landrace (Mixteco) from Oaxaca, the area of early maize diversification. Both of these maize genotypes have thrived for thousands of years with little to no nitrogen inputs and so we hypothesized that they host beneficial bacteria that allow them to thrive in nitrogen stressed conditions. We identified multiple root endophyte species from each ancient maize relative that increased the growth of annual ryegrass (model maize relative) under nitrogen starvation. Furthermore, research infers these strains were vertically transmitted to new generations of plants, potentially through seed, indicating selection pressure for Parviglumis and Mixteco to maintain them in their microbiome.

## Introduction

Maize (corn) is among the world’s top three most important food crops, with over 1 billion tonnes produced in 2016 globally (FAO, 2016). Maize is cultivated around the world with large amounts of genetic diversity (Diggle and Friedman, 2011), but phylogenetic analysis traces all modern maize (*Zea mays* ssp. *mays*) to a single domestication event that occurred in southern Mexico 9,000 years ago from its wild ancestor *Z. mays* ssp. *parviglumis* (Parviglumis teosinte) with a minor contribution from *Z. mays* ssp. *mexicana* (Matsuoka et al., 2002). Parviglumis teosinte shows tolerance to low nitrogen stress (Gaudin et al., 2011; Han et al., 2015), and as a wild plant evolved in the absence of human nitrogen inputs, unlike its cultivated progenitors. Parviglumis can still be found thriving in nutrient poor soil in the Balsas River valley of Mexico. Elegant research has revealed the genetic differences that contribute to the dramatic morphological differences between modern maize and its Parviglumis ancestor (Matsuoka et al., 2002; Doebley et al., 2006), however, there has been little study on the microbiomes of these plants.

Endophytes are plant associated microbes that live inside host plants without causing disease (Johnston-Monje and Raizada, 2011b; Hardoim et al., 2015; Brader et al., 2017). Research suggested that a large fraction of the bacterial seed endophytes from Parviglumis were no longer present in modern maize (Johnston-Monje and Raizada, 2011a). Another study suggested that many root and shoot endophytes in Parviglumis could likely be vertically transmitted to subsequent generations presumably via the seed microbiome, and that certain bacterial species would be maintained regardless of the soil they were grown in, including sterile sand (Johnston-Monje et al., 2014). The latter study showed similar results with root and shoot endophytes of Mixteco (*Z. mays* ssp. *mays* landrace Mixteco), a giant, ancient pre-Columbian landrace of maize from the state of Oaxaca in Mexico, near the site of early maize diversification, that reportedly is grown by indigenous farmers on low-nutrient soils and may represent a missing link between modern maize and Parviglumis (Matsuoka et al., 2002). Recently, another giant farmer-selected landrace from Oaxaca growing on nutrient-poor soils was shown to possess nitrogen-fixing endophytes in its above ground brace roots (Van Deynze et al., 2018). This landrace, known as Sierra Mixe, secretes large amounts of sugar rich mucilage from brace roots as the plant matures, and this creates an environment suitable for nitrogen fixation and transfer to the plant host (Van Deynze et al., 2018). Researchers have speculated that the ability of these primitive maize genotypes to thrive in nutrient poor conditions may be attributed to beneficial endophytic bacteria (Triplett, 1996; Van Deynze et al., 2018). Moreover, it has previously been hypothesized that the root microbiome of Parviglumis hosts robust nitrogen fixing endophytes that perhaps contribute to its tolerance to nitrogen stress (Han et al., 2015). We previously demonstrated that the bacterial endophytes isolated from the below ground roots of Mixteco and Parviglumis could grow *in vitro* on nitrogen-free media (Johnston-Monje and Raizada, 2011a) and speculated that they might contribute nitrogen to their host plants, i.e., that they are diazotrophs.

Modern agriculture demands vast quantities of synthetic nitrogen fertilizer to grow cereal crops such as corn, wheat, and rice, which unlike their wild relatives have been bred with high levels of synthetic nitrogen fertilizer. These grasses are essential to feeding our growing population, but the burning of natural gas to create the 100 teragrams of nitrogen fertilizer produced every year has proven unsustainable in the context of our knowledge of global warming (Galloway et al., 2003, 2004; Cheng, 2008). Therefore, there is incentive to find alternative ways to provide bioavailable nitrogen to our cereal crops, and the use of diazotrophic endophytes has shown potential (Iniguez et al., 2004; Pankievicz et al., 2015; Rosenblueth et al., 2018).

Screening for diazotrophic endophytes that can provide bioavailable nitrogen to their host plants is somewhat complicated. Screening bacteria for plant growth promoting properties *in vitro* is fast (Narula et al., 1981; Ahmad et al., 2006, 2008), however the plant environment is vastly different, and so microbial *in vitro* performance may not correspond to their *in planta* performance. At the same time, high-throughput *in vitro* experiments can act as preliminary screens to reduce sample sizes based on mechanism of action.

The gold standard for determining microbial capacity for nitrogen fixation is the acetylene reduction assay. However, this method is difficult to scale for a larger library of candidates in a high throughput screen. A simpler and more scalable alternative is to screen for microbial growth in nitrogen free media, although this method has several limitations. Both these methods do not give information about potential bacterial nitrogen transfer to plant hosts. Measuring bacterial ammonium secretion is a common method to assess nitrogen transfer from bacteria to their environment (Pankievicz et al., 2015). However, the direct product of nitrogen fixation, ammonia, is toxic to maize at locally high concentrations (Schortemeyer et al., 1997; Britto et al., 2001), whereas the downstream product, glutamine (Gln), is the compatible, primary assimilate and major transport form for external nitrogen (Magalhães et al., 1990; Hirel et al., 2001) and so may be relevant in diazotroph-maize relationships. Here, we modify an existing Gln biosensor protocol to develop a high throughput assay to identify bacteria that secrete bioavailable nitrogen in the form of Gln. This biosensor was previously validated as a measure of nitrogen fixation derived from rhizobia bacteria inhabiting legume nodules (Thilakarathna et al., 2017, 2018).

In addition to nitrogen fixation, plant associated bacteria can improve plant growth and nutrient use efficiency by modulating and secreting phytohormones such as auxins, cytokinins, and ethylene (Cassán et al., 2009; Santoyo et al., 2016). Secretion of the auxin, indole acetic acid (IAA), by root associated bacteria has been shown to alter root morphology in grasses (Dobbelaere et al., 1999). Stimulating increased root growth and surface area to improve nutrient uptake is a mechanism by which plant associated bacteria can improve nitrogen use efficiency (NUE) (Di Benedetto et al., 2017, 2019). In addition to nitrogen, bacteria can assist in the uptake of important micronutrients such as Fe, Cu, and Cd. Collectively these bacteria are classified as biofertilizers for their ability to provide and promote uptake of nutrients. Testing bacterial isolates *in planta* under nitrogen starvation would also identify these potentially useful isolates that confer tolerance to nitrogen stress by improving NUE and nutrient uptake. To develop a high-throughput *in planta* model system to identify nutrient promoting endophytes from maize, a recent study from our group assessed a diversity of forage and turf grass species (Shehata et al., 2017b). Annual ryegrass proved to be a good model and belongs to the family Poaceae along with maize, and hence they are genetic relatives. Annual ryegrass can be grown in test tubes and was shown to be highly growth/biomass responsive to nitrogen inputs, and identified a maize seed endophyte that could increase root biomass in the absence of external nitrogen (Shehata et al., 2017b).

In this study, we used the described combination of *in vitro* screens and the annual ryegrass *in planta* assay to screen the previously isolated root and shoot endophytes (Johnston-Monje et al., 2014) of the wild ancestral teosinte Parviglumis and the ancient Mexican landrace Mixteco for strains that confer tolerance to nitrogen starvation. Both of these maize genotypes thrive in nutrient poor soil with little to no N-fertilizer inputs, and this may in part be due to beneficial N-fixing and growth promoting endophytes. Altered root/shoot biomass ratio was also calculated, since plants have been shown to acclimate to low nitrogen stress by proliferating their roots to scavenge for nitrogen while limiting shoot growth and hence nitrogen demand (Gaudin et al., 2011). This study tests the hypothesis that Parviglumis and Mixteco possess nitrogen fixing endophytes that can act as nitrogen biofertilizers to promote plant growth under nitrogen stressed conditions.

## Materials and Methods

This study used two simple preliminary *in vitro* experiments to narrow the number of microbial targets we would test *in planta:* first, assessing their growth in N-free liquid media to identify candidates that can support their growth on N_2_ gas alone; and second, testing for bacterial Gln secretion on N-free agar using a previously engineered Gln biosensor (Tessaro et al., 2012). This biosensor was previously validated as a measure of nitrogen fixation derived from rhizobia bacteria inhabiting legume nodules (Thilakarathna et al., 2017, 2018); here we use a modified protocol to identify maize endophytes that can secrete this bioavailable form of nitrogen while growing on nitrogen free agar media.

### Source of Endophytes

**FIGURE 1:**
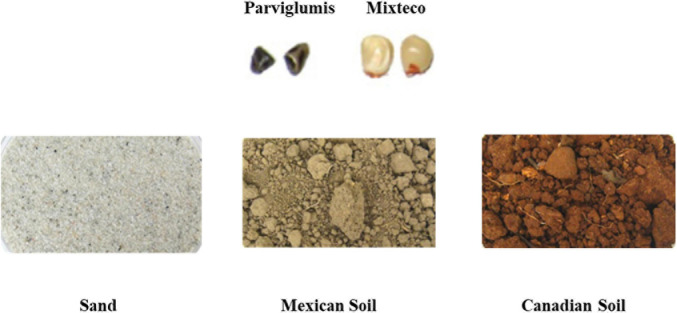
Sources of root and shoot endophytes tested in this study. Endophytes were isolated from surface sterilized roots and shoots of Parviglumis and Mixteco grown in three different soils (including sterile sand) as part of a past study (Johnston-Monje et al., 2014).

The bacterial endophytes used in this study were previously reported, with the corresponding Genbank accession numbers located within (Johnston-Monje et al., 2014). Briefly, they were isolated from surface-sterilized root and shoot tissues from the wild ancestor of modern maize, *Z. mays* ssp. *parviglumis* (Parviglumis), and an ancient Mexican landrace (*Z. mays* ssp. *mays*, landrace Mixteco), growing on three types of soil in pots in a Canadian greenhouse: a Canadian agricultural soil that has grown modern hybrids for decades; a non-agricultural Mexican soil on which Parviglumis was observed growing; and sterile sand (Figure 1).

### Endophyte Growth in Nitrogen Free Medium

Glycerol stocks of endophytes stored in 96 well plates at −80°C were used to inoculate 900 μL Burks N-free media using a 96-pin replicator. This medium consisted of (per L): 0.2 g MgSO_4_, 0.8 g K_2_HPO_4_, 0.2 g KH_2_PO_4_, 0.130 g CaSO_4_, 0.00145 g FeCl_3_, 0.000253 g Na_2_MoO_4_, and 20 g sucrose. Phosphates were autoclaved separately and cooled before mixing to avoid formation of a precipitate which would interfere with OD_600_ readings in the spectrophotometer. Plates were sealed with a sterile breathable membrane (BF-400-S, Corning) and incubated in an anaerobic chamber (Shellab Bactron IV, Sheldon Manufacturing, Inc., Cornelius, OR, United States) for 6 days at 25°C. After growth in N-free medium, cells were resuspended, and the OD_600_ was measured using a 96-well spectrophotometer (Molecular Devices, SpectraMax 384 Plus). Each endophyte strain was tested with three replicates across three different 96-well plates, with two independent trials.

### Burk’s GlnLux Gln Secretion Assay

#### Preparation of GlnLux Biosensor Cells

From a glycerol stock, single streaked GlnLux colonies grown on LB agar + carbenicillin (100 mg/ml) and kanamycin (50 mg/ml) at 37°C were used to inoculate 25 ml of LB in 50 ml tubes. LB media was supplemented with 50 μL 2 M glucose, 25 μL 0.2 M Gln, and 25 μL kanamycin and 25 μL carbenicillin. These antibiotics at these concentrations were used to supplement all GlnLux media in this report. Liquid cultures were grown overnight at 37°C with shaking at 200 rpm in Burk’s nitrogen free Burk’s media to deplete endogenous nitrogen. The cultures were centrifuged at 2,500 × *g* at 20°C for 10 min, then washed 2× in nitrogen free Burk’s media. Cells were resuspended to an OD_595_ of 1.0 in the same media.

#### Endophyte Colony Assay

The protocol was adapted from Tessaro et al. (2012). Glycerol stocks of endophytes stored in 96 well plates at −80°C were spotted on *Burk’s* GlnLux plates using a 96-pin replicator. Burk’s GlnLux plates were made as follows: per L, 800 ml of M9 medium (no NH_4_, pre-warmed to 42°C) was mixed with 100 ml of molten agar (100 g/L), cooled to 42°C, to which 100 ml of GlnLux Burk’s liquid culture (OD595 = 1.0) was added. Subsequently, 75 ml of this mixture was poured into Petri dishes (150 × 15 mm), cooled at room temperature, and stored at 4°C. Molten agar was mixed with nitrogen-free Burk’s media to a final concentration of 10 g/L and cooled to 42°C. Washed GlnLux culture in nitrogen free Burk’s media was added to the molten agar media to make 10% of the final volume. 75 ml of this mixture was poured in large Petri dishes, cooled at room temperature, and stored at 4°C. Inoculated plates were incubated at 30°C for 3 days to allow endophytes to fix nitrogen and grow. After 3 days, GlnLux plates were moved to 37°C incubator to allow the GlnLux biosensor cells to grow for 24 h. These plates were then imaged using a ChemiProHT Luminescence Imaging System (Roper, United States) with Winview 32 software with a 10-min exposure. The CCD chip was cooled using liquid nitrogen for 1 h prior to imaging to reduce background dark noise. Treatments and replicates were normalized by equating minimum and maximum light intensities across plates in the Winview 32 software. Each endophyte plate had three replicates.

### Annual Ryegrass Biofertilizer Experiment

#### Endophyte Seed Coat Preparation

Root and shoot endophytes that showed either Gln secretion or growth in N-free liquid media were selected for testing as potential nitrogen biofertilizers using annual ryegrass. The forage species annual ryegrass (*Lolium multiflorum*) was selected as the model species and used to screen a library of maize seed endophytes for candidates that could increase plant biomass in the absence of bioavailable nitrogen (Shehata et al., 2017b). Glycerol stocks stored at −80°C of selected root/shoot endophytes were inoculated on LB agar plates. The sample set of 51 candidate endophytes tested in the annual ryegrass biofertilizer experiment can be seen in Table 3. Single colonies were used to inoculate LB liquid medium and incubated at 37°C shaking at 200 rpm for 2 days. Cells were centrifuged, washed twice in 10 mM tris HCl (pH 7), then suspended to OD_595_ = 0.5 ± 0.1. From each suspension, 500 μl were diluted in 5 ml of 9.3% PVP aqueous solution. Sterilized seeds were mixed with endophyte solutions and placed on a rotary shaker for 1 h with shaking at 200 RPM to coat the seeds.

#### Plant Growth System

This consisted of glass culture tubes with 15 ml of 0.5 strength MS medium with no nitrogen (pH 5.8) (M531, Phytotech, United States). The media was supplemented (per L) with 250 μl nicotinic acid (1 mg/ml), 500 μl pyridoxine HCl (0.5 mg/ml), 5 ml thiamine HCl (100 mg/l), 500 μl glycine (2 mg/ml), and 2 g Phytagel (P8169, Sigma, United States) in double distilled water (Shehata et al., 2017b). To solidify Phytagel, 0.166 g/l CaCl_2_ and 90 mg/l MgSO_4_ were added. Seeds were soaked in 70% ethanol for 1 min then in bleach for 20 min. Seeds were then rinsed 6 times in sterile ddH_2_O.

#### Plant Growth

Seven endophyte coated seeds were planted per tube (1 replicate), and each endophyte was tested with three replicates, randomized. There were 2–3 independent trials per treatment. Plants were moved to the dark to germinate for 7 days then grown at room temperature in a 16/8 h light dark cycle under 100–120 μmol m^–2^ s^–1^ cool white fluorescent light. The large number of candidate endophyte strain treatments meant the experiment was performed in batches, each with a Tris-PVP uninoculated control for comparison. After growing for 4 weeks, plants were removed from the tubes, and the gel substrate was removed manually and by soaking in a 60°C water bath. Plants were placed between two dry paper towels for 10 s, then placed on paper to air dry for 10 min. Shoots were separated from roots, and each was weighted as a total mass per tube, divided by the number of plants per tube to calculate the mean fresh weight per experimental unit (tube).

### Statistical Methods

#### Growth in Burk’s N-Free Liquid Media Experiment

The experiment was set up in a block design with 3 blocks. The fixed effect and independent variable was the endophyte strain, and the random effect was the block. The dependent variable was growth as measured by OD_600_. The study had a type 1 error rate of α = 0.05. The statistical analysis was performed on SAS version 9.4 software using the general linearized mixed model (Glimmix) procedure with a lognormal distribution. The assumptions of this model are: normal distribution of residuals, homogenous error variance across fixed and random effects, and error that is independent of treatment effects. To test the assumption of normality, a Q–Q plot of studentized residuals was created using the UNIVARIATE procedure; the distribution was assessed for normality. In addition to the Q–Q plot, a formal test of normality was conducted (Shapiro-Wilk test). To test the assumption of homogenous error variance, scatter plots of studentized conditional residuals across the fixed effect of strain and random effect of block were created. The distribution of these was assessed to ensure there were no patterns observed in the distribution of studentized residuals. Heterogeneous error variance was not seen and so the model was not modified. The fixed effect was tested using an *F* test, and the random effect was tested using a log likelihood test. To assess outliers in the data, the studentized residuals were analyzed to see if any treatment data was beyond the envelope of ± 3.4 as outlined by Lund’s test, but no data points were removed. The mean OD_600_ growth values of each endophyte strain was compared pairwise with no adjustment. The letter values were generated using the Pdmix800 program.

#### Annual Ryegrass Biofertilizer Experiment

The experiment was a completely randomized design with 51 treatments, each with 3 replicates. The fixed effect (independent variable) was the endophyte strain, and the dependent variables were root and shoot biomass. The large sample size meant that the experiment was divided into batches, each with an uninoculated control, and batch was included as a random effect in the analysis. This exploratory study had a type 1 error rate of α = 0.1, although *P*-values < 0.05 and 0.01 were noted. Statistical analysis was performed on SAS version 9.4 software using the general linearized mixed model (Glimmix) procedure with an identity link function. The model assumes a normal distribution of residuals, homogenous error variance across fixed effects and random effects, and error that is independent of treatment effects. Normality of residuals was tested with UNIVARIATE procedure to create a Q–Q plot of studentized residuals as well as a Shapiro-Wilk test; both tests showed residuals were normally distributed. Scatter plots of studentized conditional residuals were created to test the assumptions of homogenous error variance across treatments (fixed effect) and batches (random effect). The Covariance structure did not need to be modified as there were no obvious patterns. The fixed effect was tested against the control with an *F*-test using a series of contrast statements in order to identify endophyte treatments that significantly increase plant biomass against the uninoculated controls. Outliers in the data were analyzed by looking at the studentized residuals, ensuring all were within the envelope of ±3.4 as outlined by Lund’s test.

### Whole Genome Sequencing

Candidate strains were grown on LB at 30°C overnight prior to DNA being isolated using the Norgen Bacterial Genomic DNA Isolation Kit (17900). A Nextera XT DNA sample prep kit (Illumina, San Diego) was used to create a paired-end library, and sequencing was done using Illumina Miseq. Genome assembly was completed using the CLC Genomics Workbench (10.0.1), while gene annotations were performed using the RAST genome annotation pipeline (Aziz et al., 2008). Genes in the nitrogen fixation operons were confirmed and reclassified using BLAST (Camacho et al., 2009).

## Results

### Root and Shoot Endophyte Growth in N-Free Liquid Media

**FIGURE 2:**
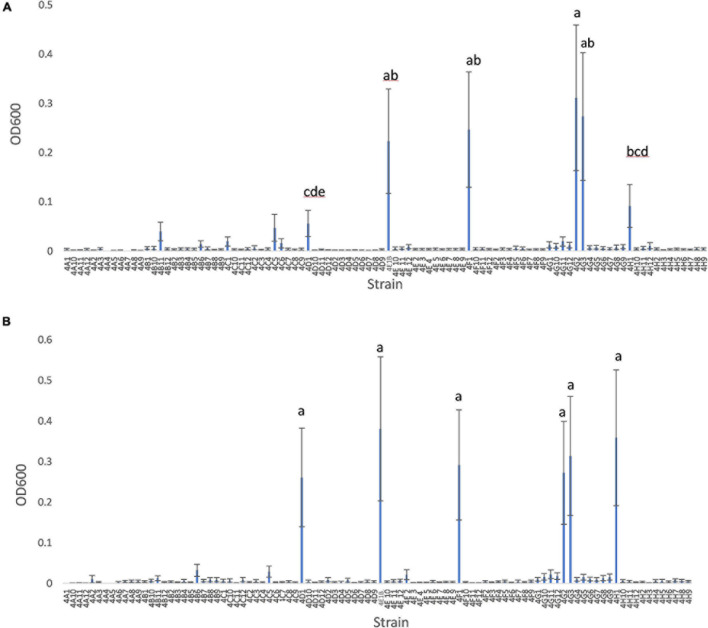
*In vitro* growth of root and shoot maize endophytes in nitrogen free medium. **(A)** Trial 1. **(B)** Trial 2. Root and shoot endophytes stored in glycerol stocks in 96-well plates were inoculated into 900 μl Burk’s N-free media in 96-deep well plates (*n* = 3). These plates were incubated anaerobically at room temperature for 6 days. After 6 days, cultures were resuspended and OD_600_ values were measured using a spectrophotometer. The error bars represent the standard error, and means with the same letter value are not statistically different.

Growing 92 maize root and shoot endophytes from Parviglumis and Mixteco in N-free Burk’s media anaerobically showed that many of these bacterial species showed modest growth or did not grow at all (Figures 2A,B). The ability to effectively colonize N-free liquid media to produce turbid cultures indicates a strain that is not limited by the nitrogen free conditions presented. There were six endophyte strains that were able to produce visibly turbid cultures and were the highest OD_600_ values seen in both trials of this experiment (Table 1). We refer to these endophytes as robust diazotrophs in this report as they can effectively colonize the N-free liquid media with only N_2_ gas to support their growth. Interestingly, all 6 of these robust diazotroph endophyte strains were isolated from root tissue (Table 1). Another point of interest is that 5 of the 6 robust strains were isolated from Parviglumis, the direct wild ancestor of modern maize (Table 1). Endophyte strains from Parviglumis and Mixteco that showed growth in N-free Burk’s media were added to the list of candidate endophytes to be tested *in planta* as nitrogen biofertilizers.

**TABLE 1:** Summary of endophytes* that grew robustly in N-free growth trials *in vitro*.

Endophyte	Host genotype	Tissue	Soil	Mean OD600
**Trial 1**				
4G2 – *Klebsiella*	Parviglumis	Root	Canada	0.311 a
4G3 – *Klebsiella*	Parviglumis	Root	Canada	0.273 ab
4F1 – *Klebsiella*	Parviglumis	Root	Canada	0.247 ab
4E1B – *Klebsiella*	Parviglumis	Root	Sterile sand	0.223 ab
4H1 – *Klebsiella*	Parviglumis	Root	Mexico	0.092 bcd
4D1 – *Bacillus*	Mixteco	Root	Mexico	0.056 cde
**Trial 2^∧^**				
4G2 – *Klebsiella*	Parviglumis	Root	Canada	0.272 a
4G3 – *Klebsiella*	Parviglumis	Root	Canada	0.314 a
4F1 – *Klebsiella*	Parviglumis	Root	Canada	0.291 a
4E1B – *Klebsiella*	Parviglumis	Root	Sterile sand	0.381 a
4H1 – *Klebsiella*	Parviglumis	Root	Mexico	0.359 a
4D1 – *Bacillus*	Mixteco	Root	Mexico	0.261 a

*Both root and shoot endophytes were screened, and shown are those that showed the highest growth in both trials.

*^∧^These six endophytes were not statistically different from one another in Trial 2.*

### Ability for Endophytes to Secrete Gln *in vitro*

**FIGURE 3:**
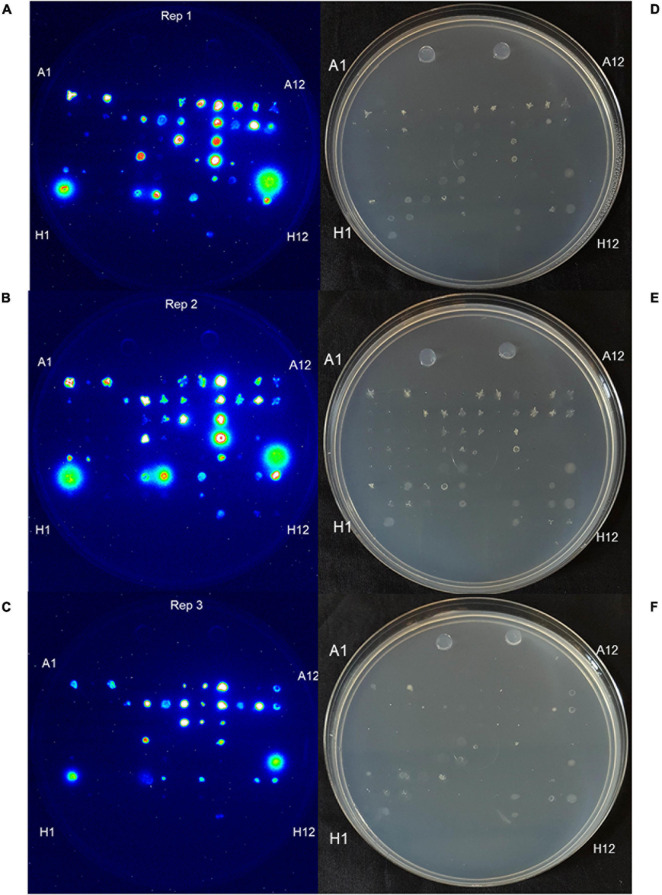
Burk’s GlnLux colony assay to screen the maize endophytes for Gln secretion *in vitro* on nitrogen-free Burk’s agar. **(A–C)** Shown is luminescence imaging of three replicates: **(A)** replicate 1, **(B)** replicate 2, and **(C)** replicate 3 of endophytes from Parviglumis and Mixteco that could secrete Gln on N-free agar as indicated by a luminescence signal. **(D–F)** Corresponding light images of these plates: **(D)** replicate 1, **(E)** replicate 2, and **(F)** replicate 3. Some strains did not grow on this N-free agar as expected.

The Burk’s GlnLux colony assay identified many root and shoot endophytes from Parviglumis and Mixteco that could grow on Burk’s N-free agar and secrete Gln to support the growth of GlnLux cells as indicated by emission of luminescence (Figure 3). In total, 34/92 of the tested root/shoot endophytes secreted Gln on N-free agar in at least 2 out of 3 independent trials. These endophytes were added to the pool of candidates to test as nitrogen biofertilizers, given their ability to secrete this plant-available form of nitrogen. The summary of this assay is shown in Table 2.

**TABLE 2:** Summary of maize endophytes that secrete Gln *in vitro* on nitrogen-free Burk’s GlnLux agar.

Root/shoot endophytes					

Position	Host	Tissue	Soil	Genus	Positive reps
4A1	Mixteco	Shoot	Sand	*Microbacterium*	3
4A12	Mixteco	Shoot	Sand	*Stenotrophomonas*	3
4A3	Mixteco	Shoot	Sand	*Microbacterium*	3
4A7	Mixteco	Shoot	Sand	*Bacillus*	3
4A8	Mixteco	Shoot	Sand	*Cellulosimicrobium*	3
4A9	Mixteco	Shoot	Sand	*Stenotrophomonas*	3
4B5	Mixteco	Root	Sand	*Microbacterium*	3
4B6	Mixteco	Root	Sand	*Enterobacter*	3
4B7	Mixteco	Root	Sand	*Stenotrophomonas*	3
4B9	Mixteco	Shoot	Canada	*Stenotrophomonas*	3
4B10	Mixteco	Shoot	Canada	*Paenibacillus*	3
4B11	Mixteco	Shoot	Canada	*Stenotrophomonas*	3
4B12	Mixteco	Root	Canada	*Stenotrophomonas*	3
4C7	Mixteco	Root	Mexico	*Agrobacterium*	3
4C9	Mixteco	Root	Mexico	*Xanthomonas*	3
4D5	Mixteco	Root	Mexico	*Paenibacillus*	3
4D9	Parviglumis	Root	Sand	*Bacillus*	3
4E9	Parviglumis	Root	Sand	*Enterobacter*	3
4E12AB	Parviglumis	Shoot	Canada	*Bacillus*(A), *Pantoea*(B)	3
4F1	Parviglumis	Root	Canada	*Klebsiella*	3
4F5	Parviglumis	Root	Canada	*Microbacterium*	3
4F6	Parviglumis	Root	Canada	*Paenibacillus*	3
4F8	Parviglumis	Root	Canada	*Enterobacter*	3
4F11	Parviglumis	Root	Canada	*Microbacterium*	3
4F12	Parviglumis	Root	Canada	*Stenotrophomonas*	3
4G7	Parviglumis	Shoot	Mexico	*Paenibacillus*	3
4A10	Mixteco	Root	Sand	*Bacillus*	2
4A11	Mixteco	Root	Sand	*Stenotrophomonas*	2
4B4	Mixteco	Root	Sand	*Microbacterium*	2
4E1AB	Parviglumis	Root	Sand	*Bacillus*(A), *Klebsiella*(B)	2
4E2	Parviglumis	Root	Sand	*Bacillus*	2
4B8	Mixteco	Root	Sand	*Enterobacter*	2
4C6	Mixteco	Root	Mexico	*Enterobacter*	2
4C8	Mixteco	Root	Mexico	*Stenotrophomonas*	1
4D8	Parviglumis	Shoot	Sand	*Stenotrophomonas*	1
4D10	Parviglumis	Root	Sand	*Pantoea*	1

### Ability of Candidate Maize Endophytes to Promote Growth of Annual Ryegrass in the Absence of Any Nitrogen Fertilizer

The *in vitro* assays above identified root and shoot endophytes from maize that could either grow in N-free liquid media and/or secrete Gln on N-free agar (Figures 2, 3), both of which are potential plant growth promoting mechanisms. To identify endophytes that have the potential to either increase NUE or provide bioavailable nitrogen to their plant hosts, we conducted an experiment testing a total of 51 candidate diazotrophs/Gln secretors for biomass effects in annual ryegrass following endophyte seed coating and growth in N-free media. It is important to note that some endophytes were excluded from this experiment if they showed poor growth in LB media which limited their potential as inoculants. Furthermore, certain wells in the 96 well endophyte source plate were each shown to have more than one colony morphology upon streaking, and so these mixed cultures were separated prior to ryegrass testing and given a letter suffix (e.g., A,B) (taxonomic confirmations for some strains are pending, F2018). Endophyte strain *Enterobacter* 3D9 was shown to increase ryegrass biomass in a past experiment (Shehata et al., 2017b; Dumigan et al., 2018), and hence it was included as a positive control in Trial 1 for comparison (from 4 independent batches). Given the large number of strains to be tested, the experiment was broken up into batches where a subset of endophytes was tested in parallel with an uninoculated negative control (endophyte buffer), and the control was included in each batch; batches were separated by time (see section “Materials and Methods”). All candidate endophytes were tested by seed coating in two independent trials; a third trial was conducted in parallel to Trial 2 on a select group of endophytes, in part based on early promising results.

**FIGURE 4:**
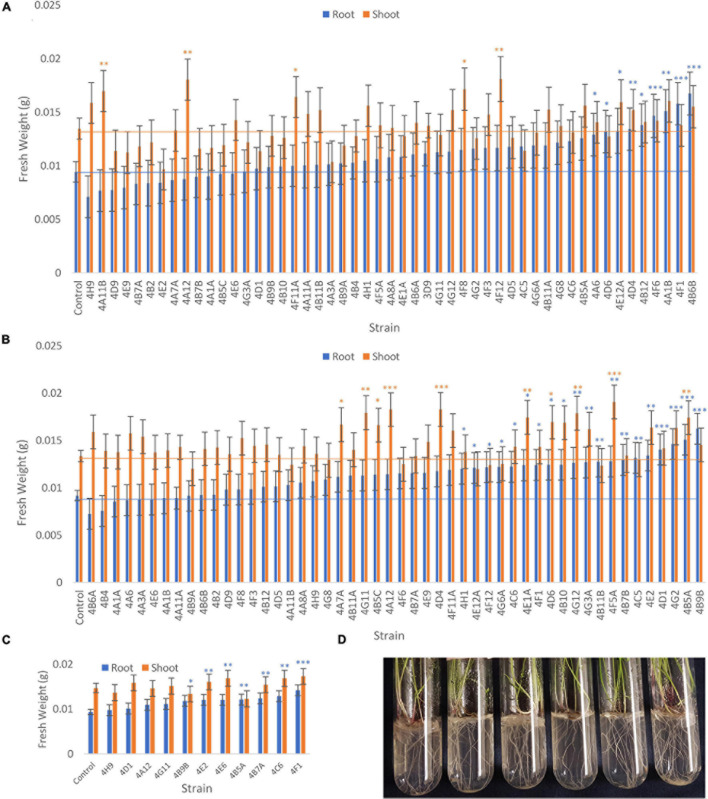
Effect of endophyte seed coating on root and shoot fresh weight of annual ryegrass. Shown are the mean fresh weights of annual ryegrass, seed-coated with endophyte strains, and grown in N-free media for 4 weeks: **(A)** Trial 1, **(B)** Trial 2, and a subset in **(C)** Trial 3. Data is ordered by increasing root biomass (blue bars), and values significantly greater than the uninoculated control are denoted by ^∗^*P* < 0.1, ^∗∗^*P* < 0.05, and ^∗∗∗^*P* < 0.01 as determined by *F*-tests. Error bars represent the standard error. **(D)** Images of the assay system: annual ryegrass growing in glass tubes in Phytagel based medium.

**FIGURE 5:**
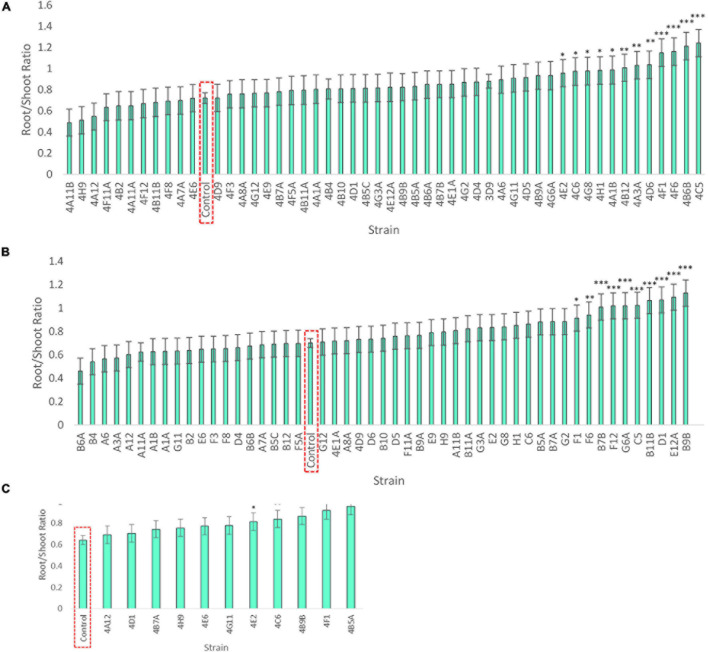
Root/shoot biomass ratio of annual ryegrass following endophyte seed treatment. Shown is the data following 4 weeks of plant growth on N-free media for: **(A)** Trial 1, **(B)** Trial 2, and a subset in **(C)** Trial 3. The uninoculated control is outlined in red, and the data is ordered by increasing root/shoot ratio. Values significantly higher than the respective negative control are denoted by ^∗^*P* < 0.1, ^∗∗^*P* < 0.05, and ^∗∗∗^*P* < 0.01 as determined by *F*-tests. Error bars represent the standard error.

The fresh weights of annual ryegrass roots and shoots for all trials following seed inoculation are shown (Figure 4, and Supplementary Tables 1–3) in addition to changes in the root/shoot biomass ratio known to be involved in plant acclimation to nutrient stress (Figure 5). The change in percentage root or shoot biomass was calculated relative to the respective mean negative control from all batches of that trial (Table 3). The screen identified four strains that increased annual ryegrass biomass when seed coated and grown in N-free media in at least two independent trials with a *P*-value less than 0.05: *Klebsiella* 4F1, *Microbacterium* 4B5A, *Bacillus* 4E2 and *Stenotrophomonas* 4A12 (Table 3):

**TABLE 3:** Summary of percent increases in annual ryegrass biomass following endophyte seed treatment compared to the respective uninoculated buffer control.

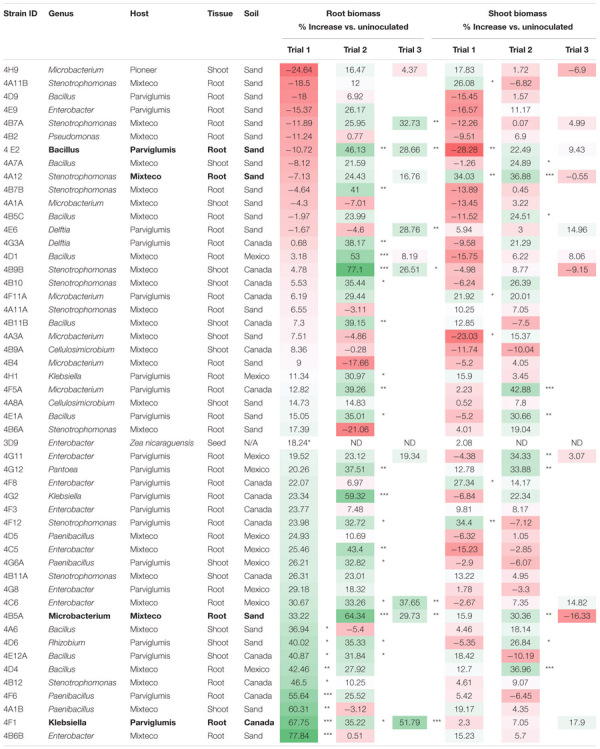

*Percent increases vs. uninoculated control values are shown as a heat map from red to green as values increase. Significant changes were calculated using an *F*-test with *P*-values of: *P < 0.1, **P < 0.05, and ***P < 0.01. Bold values indicate strains that significantly improve biomass across trials.

*Klebsiella* 4F1 increased root biomass compared to the uninoculated control by 67.8% (*p* = 0.0012), 35.2% (*p* = 0.056), and 51.79% (*p* = 0.0008) in Trials 1, 2, and 3, respectively (Table 3 and Figure 4). This endophyte significantly increased the root to shoot ratio in two independent trials (Figure 5) and did not significantly increase shoot biomass in either trial. *Microbacterium* 4B5A significantly increased root biomass by 64.3 and 29.7% in Trials 2 and 3, respectively (*p* = 0.0006, 0.041), however showed a non-significant increase of 33.2% in Trial 1 (*p* = 0.1129) (Table 3 and Figure 4). This candidate endophyte significantly increased the root/shoot biomass ratio in only one trial (Figure 5). *Bacillus* 4E2 did not increase root biomass in the first trial, however, in two subsequent independent trials this candidate endophyte significantly increased root biomass by 46.13% (*p* = 0.0128) and 28.66% (*p* = 0.0483), respectively. This candidate endophyte did not significantly increase the root/shoot ratio in any trial (*p* > 0.05) (Figure 5). *Stenotrophomonas* 4A12 showed significant increases in shoot biomass compared to uninoculated controls in both trials, with a 34.0% increase in Trial 1 (*p* = 0.0108) and a 36.9% increase in Trial 2 (*p* = 0.0082) (Table 3 and Figure 4). Surprisingly, a third independent trial of 4A12A showed a non-significant decrease of 0.5% in shoot biomass compared to the negative control (Table 3). The root to shoot ratio of 4A12 coated ryegrass did not significantly change in any trial compared to the negative control (Figure 5).

Only endophytes with significant increases in plant biomass were considered interesting in these initial high-throughput *in planta* screens. It is important to note that there are many endophyte seed treatments that significantly increased root or shoot biomass compared to the control in a single trial, however, only endophyte seed treatments that gave a consistent result are highlighted in this Results text section. All biomass increases can be seen on the graphs (Figure 4) or in the summary (Table 3).

### Whole Genome Sequencing of Top Microbial Candidates

Four strains were selected for whole genome sequencing based on their performance at improving biomass of annual ryegrass under nitrogen starvation. Strain 4F1 was identified as belonging to the species complex *Klebsiella pneumoniae-variicola*, and its genome was predicted to encode 19 *nif* open reading frames (ORFs) involved in nitrogen fixation (Supplementary Figure 1). Nitrogenase genes were not found in the other candidate strains. Strain 4E2 was identified as belonging to the species complex *Bacillus velezensis-amyloliquefaciens.* Strain 4A12 was identified as *Stenotrophomonas indicatrix.* Stain 4B5A was identified as *Microbacterium barkeri*.

## Discussion

Organisms including plants can adapt to stressful environments by adopting beneficial bacteria in their microbiomes (Johnston-Monje and Raizada, 2011b; Hardoim et al., 2015; Mousa et al., 2015, 2016; Brader et al., 2017; Shehata et al., 2017a; Van Deynze et al., 2018). Nitrogen (N) is a vital plant macronutrient needed for protein, DNA, and chlorophyll biosynthesis, therefore nitrogen stress is critically damaging to growing plants. Today, modern maize is grown with large amounts of synthetic N-fertilizer inputs, and breeding efforts in maize over the past several decades have focused on increasing yield responses to increasing N supply (Neill et al., 2004; Kant et al., 2011). Research has suggested that the domestication of maize caused a community shift in the rhizosphere microbiomes, in comparisons of modern hybrids to ancestral teosintes (Brisson et al., 2019). Interestingly, the largest shift in the plant-microbe community structure appears to be from the domestication of teosinte to landraces, while modern agronomic practices show the largest effect on potential microbe-microbe interactions (Schmidt et al., 2020).

A past study from our group showed that the seed microbiomes of 14 wild and domestic maize varieties have undergone significant changes over the course of evolution, and subsequent human selection and migration in the Americas (Johnston-Monje and Raizada, 2011a). The study further showed that approximately half of the endophytes in the direct, wild ancestor of modern maize, Parviglumis, are no longer present in modern maize. It is possible that humans have selected against the retention of beneficial plant bacteria that assist wild maize to grow in the absence of human-derived nitrogen. Therefore, in this study we tested root and shoot microbes from Parviglumis. We also tested endophytes from an ancient cultivated landrace from Mexico called Mixteco (Johnston-Monje and Raizada, 2011a). Parviglumis and Mixteco both originate from southern Mexico: Parviglumis was domesticated in the Balsas River Valley (Matsuoka et al., 2002; Piperno et al., 2009; Ranere et al., 2009), while the Mixteco accession used in this study (CIMMYT: OAX 569) was collected from the Nochixtlán District in the Mixteca Region of the nearby state of Oaxaca, the region of early maize diversification (Matsuoka et al., 2002). Critically for this study, in the Mixteca region, maize is cultivated by the indigenous Mixtec peoples on acidic, low-nitrogen soils, often on steep, depleted hillsides (Vergara-Sánchez et al., 2005; Bautista-Cruz et al., 2014), and despite such nitrogen limitation, Mixteco maize is giant in stature. Acidic soils prevent plant availability of most macronutrient fertilizers including nitrogen. The Nochixtlán Valley has been in continuous settlement since 2000 BC, and was the geographic center of the Mixtec peoples from 700 BC to 1600 AD, having a population estimated to be 50,000 people when the Spaniards arrived (Spores, 1969). The Nochixtlán Valley is only 50 km away from where another maize landrace called Sierra Mixe was collected (selected by the adjacent indigenous Mixe-speaking people) and recently reported to host potent nitrogen fixation in mucilage secreted from its above ground brace roots (Van Deynze et al., 2018). We thus hypothesized that the nitrogen stressed conditions of these ancient maize relatives may have driven natural and human selection, in Parviglumis and Mixteco respectively, for beneficial relationships with endophytic bacteria that provide tolerance to nitrogen starvation. We used three complementary approaches, two *in vitro* and one *in planta*:

Initially we tested the ability of 92 root and shoot endophytes of Parviglumis and Mixteco to grow in N-free liquid media, suggestive of N-fixation *in vitro*. Many endophytes showed minor levels of growth, however, there were six endophyte species that consistently grew to the highest OD_600_ values in both trials (Figure 2). Though 55% of the root and shoot endophyte library was comprised of endophytes isolated from Parviglumis, with 45% from Mixteco (Johnston-Monje et al., 2014), five out of the six robust growing diazotrophs were from the former (Parviglumis) (Figure 2). All six of the endophytes were isolated from surface-sterilized root tissue, which comprised 75% of the library (Johnston-Monje et al., 2014). All endophyte strains from Mixteco and Parviglumis that could grow in N-free liquid were added to the pool of candidates to be tested as biofertilizers in annual ryegrass.

Glutamine is the downstream assimilate of fixed nitrogen that also acts as a primary transport form of nitrogen in maize (Hirel et al., 2001; Näsholm et al., 2009; Tegeder and Rentsch, 2010), therefore, it has been previously hypothesized that, unlike rhizobia (in the legume symbiosis), Gln may be a nitrogen containing metabolite secreted by endophytes for cereal crops to utilize (Allaway et al., 2000). Therefore, in our second assay, we screened root and shoot endophytes from Mixteco and Parviglumis for their ability to grow on Burk’s N-free agar and secrete Gln to support the growth of adjacent GlnLux biosensor cells and emit luminescence (Figure 3). The screen showed that 34/92 of the tested endophytes could grow and secrete Gln, which would be highly bioavailable to maize. These strains were also added to the pool of candidates to be tested as nitrogen biofertilizers in annual ryegrass.

Finally, in the third assay, the ability of the selected candidate endophytes to promote the growth of annual ryegrass in N-free media was tested. Annual ryegrass is a heterologous host, and hence the numbers might be greater in the native hosts. Four strains were found to significantly increase plant biomass in independent trials, and many more showed significant or borderline increases in at least one trial (Figure 4 and Table 3). The 4 strains were 4F1, 4A12, 4E2, and 4B5A and originate equally from Mixteco and Parviglumis. All 4 of these strains were isolated as root endophytes. As research has suggested that a substantial portion of the juvenile maize rhizosphere originates from the plant (Johnston-Monje et al., 2016) including Parviglumis and Mixteco (Johnston-Monje et al., 2014), it may be that some of the beneficial activities of the root endophytes may actually be taking place in the surrounding soil. Interestingly 3 of the 4 endophytes that confer tolerance to N-starvation were originally isolated from plants grown in sterile sand in a greenhouse, suggesting they originated from seeds rather than being acquired from the soil (Johnston-Monje et al., 2014) which would be consistent with our hypothesis of long term natural and human selection on maize. Indeed, nitrogen fixing *K. pneumoniae-variicola* has been previously isolated from the seed endosphere of Parviglumis (Supplementary Table 4), suggesting this maize hosts nitrogen fixing bacteria that are seed borne, supporting the hypothesis that the strains in this study may have been vertically inherited. Additionally, these *Klebsiella* species that demonstrated nitrogen fixation also secreted Gln as demonstrated by the GlnLux biosensor assay (Supplementary Table 4). Furthermore, our research has shown that (seed-derived) endophytes can be deposited into the rhizosphere and root surfaces (Johnston-Monje et al., 2016; Mousa et al., 2016; Shehata et al., 2017a) where some of the above activities would need to be localized. Combined, the results of the three complementary assays used in this study provide evidence that the wild ancestor of modern maize, and an ancient race of corn from the region of early maize diversification, possess endophytes with the potential to confer plant tolerance to nitrogen stress.

As noted, four endophyte strains consistently promoted the growth of annual ryegrass in the absence of nitrogen fertilizer. *K. pneumoniae-variicola* 4F1 was isolated from surface sterilized root tissue of Parviglumis and belongs to the genus *Klebsiella*. *K. pneumoniae-variicola* 4F1 significantly increased both root biomass and the root/shoot biomass ratio of annual ryegrass compared to the uninoculated control in trials 1 and 2 (Figures 4, 5). An increase in the root to shoot ratio is a conserved adaptation among plant species in response to nitrogen stress, as plants limit resource allocation to shoot tissue to favor of roots which have the ability to scavenge soil for nitrogen – a trait that we have previously characterized in Parviglumis (Gaudin et al., 2011). Interestingly, Parviglumis increases its crown root length 285% in response to low nitrogen conditions compared to a 30% elongation seen in a modern inbred maize line (Gaudin et al., 2011). Such an adaptation likely contributes to the tolerance to nitrogen stress seen in Parviglumis, and perhaps may be, in part, attributed to beneficial root endophytes such as *K. pneumoniae-variicola* 4F1. This strain was also found to grow well in nitrogen free media (Figure 2) and possess 19 ORFs for nitrogen fixation in its genome (Supplementary Figure 1) which together suggest the capacity for nitrogen fixation. Our previous study showed that this strain will also produce acetoin *in vitro* (Johnston-Monje et al., 2014), a compound that can alter plant hormone production to increase growth (Hu et al., 2003), and so the observed effect on plant growth may be a combination of mechanisms.

*Bacillus velezensis-amyloliquefaciens* 4E2 increased root biomass in annual ryegrass in two out of three trials. As above, *Bacillus* 4E2 was originally isolated from surface sterilized Parviglumis roots grown in sterilized sand, suggesting this endophyte is vertically transmitted to the next generation of plants (Johnston-Monje et al., 2014). The genus *Bacillus* is a well-studied plant growth promoting bacteria and has been shown to promote plant growth by a variety of mechanisms (Hu et al., 2003). This genus of spore-forming bacteria has been commercialized into a wide variety of biostimulants and biopesticides, and so it is unsurprising to find a plant growth promoting strain endophytic strain in the roots of Parviglumis plants. Species of *Bacillus* have been shown to improve NUE in greenhouse and field conditions by improving nutrient uptake (Adesemoye et al., 2008, 2009). Interestingly, the whole genome sequence of *B. velezensis-amyloliquefaciens* 4E2 did not identify any nitrogenase genes, suggesting an alternative mechanism for the observed growth promotion under nitrogen starvation. The strain did however secrete Gln on N-free agar (Figure 3), suggesting potential nitrogen transfer to its plant host. It could be that this strain is secreting or regulating phytohormones that are stimulating root growth and improving nitrogen/nutrient use efficiency when its host plant is under nitrogen stress. Again, it is interesting to speculate whether favorable selection pressure has caused Bacillus species such as 4E2 to be passed down to the next generation of plants as a member of a healthy Parviglumis microbiome to confer some level of tolerance to nitrogen stress.

*Stenotrophomonas indicatrix* 4A12 was found to significantly increase shoot biomass in trials 1 and 2 (Figure 4). The strain did not grow in Burk’s N-free liquid media in either trial (Figure 2), however, it did grow on Burk’s N-free agar and secrete Gln (Figure 3). Whole genome sequencing of 4A12 did not identify any nitrogenase genes in the annotation. Taken together, it was surprising that the endophyte did not increase root biomass in any trial (Figure 5), since the standard response to nitrogen stress in plants, as already noted, is to reallocate carbon from the shoots to the roots to promote growth (Han et al., 2015). The mechanism by which 4A12 is stimulating shoot growth is not currently known. The previous study from where this isolate originates found that it solubilized phosphate and produced acetoin *in vitro* (Johnston-Monje et al., 2014). *S. indicatrix* 4A12 could be stimulating annual ryegrass shoot growth under nitrogen starvation by secreting bioavailable Gln, stimulating plant growth by phytohormone regulation, and/or improving uptake of nitrogen and other nutrients such as phosphate. It should be noted that the host genotype of this endophyte is Mixteco which possesses a giant shoot, but whether *S. indicatrix* 4A12 has any causal relationship to this gigantism awaits further testing. *S. indicatrix* 4A12 was isolated from surface sterilized root tissue that were grown in sterilized sand, meaning this endophyte was likely vertically transmitted from parental Mixteco plants rather than incorporated from the surrounding soil (Johnston-Monje et al., 2014). A previous study demonstrated that endophytes of the genus *Stenotrophomonas* were widespread in the seeds of a diversity of maize genotypes (unpublished data).

*Microbacterium barkeri* 4B5A significantly increased root biomass in 3 independent trials, and showed some of the largest biomass increases observed compared to uninoculated control plants (Figure 4). There have been reports of nitrogenase activity in *Microbacterium* (Gtari et al., 2012), but to our knowledge no reports on endophytic N-fixing *Microbacterium* species. *M. barkeri* 4B5A did not show growth in N-free media (Figure 2), and nitrogenase genes were not found in the whole genome sequence. However a past study demonstrated that this endophyte secretes acetoin (Johnston-Monje et al., 2014), and so is likely promoting plant growth under nitrogen starvation by regulating plant hormones, and/or improving nutrient and NUE rather than through nitrogen fixation. As with *S. indicatrix* 4A12, this endophyte was isolated from surface sterilized Mixteco roots grown in sterile sand, again suggesting vertical transmission.

As this study was an exploratory high throughput screen looking at biomass increases, the underlying mechanisms were not examined and likely vary between endophytes. The main conclusion that can be drawn from the *in planta* experiments is that under nitrogen stress, these seed coated endophytes have biofertilizer activity and will increase root or shoot biomass of their heterologous annual ryegrass host (Figure 5). The endophytes may increase host NUE as discussed earlier or contribute nitrogen and/or other nutrients to their plant host. The data supports the hypothesis that Parviglumis and Mixteco possess endophytes that can act as nitrogen biofertilizers to promote plant growth under nitrogen starvation. Future experiments involving bacterial gene knockouts are needed to clarify the underlying mechanisms for the increase in plant biomass under N starvation conditions. Additionally, these bacterial isolates should be tested in larger scale experiments using maize.

## Data Availability Statement

The datasets presented in this study can be found in online repositories. The names of the repository/repositories and accession number(s) can be found below: https://www.ncbi.nlm.nih.gov/genbank/, JF776463–JF776567.

## Author Contributions

CD and MR designed the study. CD, JM, and JG conducted the *in planta* assays. CD conducted all the *in vitro* assays in the manuscript and performed the statistical analysis. AS performed the 16S sequencing to confirm bacterial genus taxonomy. OH facilitated the bacterial whole genome sequencing. All authors contributed to the article and approved the submitted version.

## Conflict of Interest

The authors declare that the research was conducted in the absence of any commercial or financial relationships that could be construed as a potential conflict of interest.

## Publisher’s Note

All claims expressed in this article are solely those of the authors and do not necessarily represent those of their affiliated organizations, or those of the publisher, the editors and the reviewers. Any product that may be evaluated in this article, or claim that may be made by its manufacturer, is not guaranteed or endorsed by the publisher.
