# Supplementary material for: Ancient Relatives of Modern Maize From the Center of Maize Domestication and Diversification Host Endophytic Bacteria That Confer Tolerance to Nitrogen Starvation
Source: Front Plant Sci. 2021 Sep 16;12:660673. doi: 10.3389/fpls.2021.660673 (PMC8485183; doi:10.3389/fpls.2021.660673)
Supplement: Supplementary file 1 [file Data_Sheet_1.docx]

# Supplementary Materials

**Supplementary Table 1. Root and Shoot Mean Values Trial 1**

| Strain Least Squares Means Trial 1 | | | | | | | | | | | |
| --- | --- | --- | --- | --- | --- | --- | --- | --- | --- | --- | --- |
| Root Biomass | |  |  |  |  | **Shoot Biomass** | |  |  |  |  |
| strain | **Estimate** | **SE** | **%Increase vs Control** |  | **P Value vs Control** | **Strain** | **Estimate** | **SE** | **%Increase vs Control** |  | **P Value vs Control** |
| Control | 0.009413 | 0.000949 |  |  |  | **Control** | 0.01346 | 0.000985 |  |  |  |
| 4H9 | 0.007094 | 0.001944 | -24.64 |  | 0.2106 | **4H9** | 0.01586 | 0.001908 | 17.83 |  | 0.1792 |
| 4A11B | 0.007672 | 0.001944 | -18.50 |  | 0.3466 | **4A11B** | 0.01697 | 0.001908 | 26.08 | * | 0.05 |
| 4D9 | 0.007719 | 0.001981 | -18.00 |  | 0.3799 | **44D9** | 0.01138 | 0.001946 | -15.45 |  | 0.2628 |
| 4E9 | 0.007966 | 0.001981 | -15.37 |  | 0.4532 | **4E9** | 0.01123 | 0.001946 | -16.57 |  | 0.2297 |
| 4B7A | 0.008294 | 0.001944 | -11.89 |  | 0.5449 | **4B7A** | 0.01181 | 0.001908 | -12.26 |  | 0.3528 |
| 4B2 | 0.008355 | 0.002122 | -11.24 |  | 0.6113 | **4B2** | 0.01218 | 0.002095 | -9.51 |  | 0.5262 |
| 4E2 | 0.008404 | 0.001944 | -10.72 |  | 0.585 | **4E2** | 0.009653 | 0.001908 | -28.28 | ** | 0.0333 |
| 4A7A | 0.008649 | 0.001981 | -8.12 |  | 0.6917 | **4A7A** | 0.01329 | 0.001946 | -1.26 |  | 0.9256 |
| 4A12 | 0.008742 | 0.001944 | -7.13 |  | 0.7161 | **4A12** | 0.01804 | 0.001908 | 34.03 | ** | 0.0108 |
| 4B7B | 0.008976 | 0.001944 | -4.64 |  | 0.8127 | **4B7B** | 0.01159 | 0.001908 | -13.89 |  | 0.2931 |
| 4A1A | 0.009008 | 0.002122 | -4.30 |  | 0.8455 | **4A1A** | 0.01165 | 0.002095 | -13.45 |  | 0.3704 |
| 4B5C | 0.009228 | 0.001981 | -1.97 |  | 0.9234 | **4B5C** | 0.01191 | 0.001946 | -11.52 |  | 0.4031 |
| 4E6 | 0.009256 | 0.001944 | -1.67 |  | 0.9322 | **4E6** | 0.01426 | 0.001908 | 5.94 |  | 0.6555 |
| 4G3A | 0.009477 | 0.001981 | 0.68 |  | 0.9735 | **4G3A** | 0.01217 | 0.001946 | -9.58 |  | 0.4864 |
| 4D1 | 0.009712 | 0.001981 | 3.18 |  | 0.8767 | **4D1** | 0.01134 | 0.001946 | -15.75 |  | 0.2545 |
| 4B9B | 0.009863 | 0.001944 | 4.78 |  | 0.8078 | **4B9B** | 0.01279 | 0.001908 | -4.98 |  | 0.7044 |
| 4B10 | 0.009934 | 0.001981 | 5.53 |  | 0.7869 | **4B10** | 0.01262 | 0.001946 | -6.24 |  | 0.6489 |
| 4F11A | 0.009996 | 0.001944 | 6.19 |  | 0.7524 | **4F11A** | 0.01641 | 0.001908 | 21.92 | * | 0.0985 |
| 4A11A | 0.01003 | 0.002122 | 6.55 |  | 0.7668 | **4A11A** | 0.01484 | 0.002095 | 10.25 |  | 0.4955 |
| 4B11B | 0.0101 | 0.002122 | 7.30 |  | 0.7409 | **4B11B** | 0.01519 | 0.002095 | 12.85 |  | 0.394 |
| 4A3A | 0.01012 | 0.001981 | 7.51 |  | 0.7136 | **4A3A** | 0.01036 | 0.001946 | -23.03 | * | 0.0966 |
| 4B9A | 0.0102 | 0.001944 | 8.36 |  | 0.6718 | **4B9A** | 0.01188 | 0.001908 | -11.74 |  | 0.3725 |
| 4B4 | 0.01026 | 0.001505 | 9.00 |  | 0.5527 | **4B4** | 0.01276 | 0.0015 | -5.20 |  | 0.6118 |
| 4H1 | 0.01048 | 0.001981 | 11.34 |  | 0.5795 | **4H1** | 0.0156 | 0.001946 | 15.90 |  | 0.251 |
| 4F5A | 0.01062 | 0.002122 | 12.82 |  | 0.5628 | **4F5A** | 0.01376 | 0.002095 | 2.23 |  | 0.8841 |
| 4A8A | 0.0108 | 0.002122 | 14.73 |  | 0.506 | **4A8A** | 0.01353 | 0.002095 | 0.52 |  | 0.9734 |
| 4E1A | 0.01083 | 0.001981 | 15.05 |  | 0.4623 | **4E1A** | 0.01276 | 0.001946 | -5.20 |  | 0.706 |
| 4B6A | 0.01105 | 0.002015 | 17.39 |  | 0.406 | **4B6A** | 0.014 | 0.00198 | 4.01 |  | 0.7756 |
| 3D9 | 0.01113 | 0.001121 | 18.24 | * | 0.0833 | **3D9** | 0.01374 | 0.001144 | 2.08 |  | 0.7696 |
| 4G11 | 0.01125 | 0.001981 | 19.52 |  | 0.3409 | **4G11** | 0.01287 | 0.001946 | -4.38 |  | 0.7511 |
| 4G12 | 0.01132 | 0.001981 | 20.26 |  | 0.3246 | **4G12** | 0.01518 | 0.001946 | 12.78 |  | 0.3558 |
| 4F8 | 0.01149 | 0.002015 | 22.07 |  | 0.29 | **4F8** | 0.01714 | 0.00198 | 27.34 | * | 0.0537 |
| 4G2 | 0.01161 | 0.001981 | 23.34 |  | 0.256 | **4G2** | 0.01254 | 0.001946 | -6.84 |  | 0.62 |
| 4F3 | 0.01165 | 0.001981 | 23.77 |  | 0.2466 | **4F3** | 0.01478 | 0.001946 | 9.81 |  | 0.4798 |
| 4F12 | 0.01167 | 0.002122 | 23.98 |  | 0.2785 | **4F12** | 0.01809 | 0.002095 | 34.40 | ** | 0.0235 |
| 4D5 | 0.01176 | 0.001981 | 24.93 |  | 0.2246 | **4D5** | 0.01261 | 0.001946 | -6.32 |  | 0.6467 |
| 4C5 | 0.01181 | 0.001981 | 25.46 |  | 0.2154 | **4C5** | 0.01141 | 0.001946 | -15.23 |  | 0.2689 |
| 4G6A | 0.01188 | 0.002122 | 26.21 |  | 0.2379 | **4G6A** | 0.01307 | 0.002095 | -2.90 |  | 0.8444 |
| 4B11A | 0.01189 | 0.002122 | 26.31 |  | 0.2345 | **4B11A** | 0.01524 | 0.002095 | 13.22 |  | 0.3811 |
| 4G8 | 0.01216 | 0.002015 | 29.18 |  | 0.1627 | **4G8** | 0.0137 | 0.00198 | 1.78 |  | 0.8999 |
| 4C6 | 0.0123 | 0.001981 | 30.67 |  | 0.1356 | **4C6** | 0.0131 | 0.001946 | -2.67 |  | 0.8439 |
| 4B5A | 0.01254 | 0.002015 | 33.22 |  | 0.1129 | **4B5A** | 0.0156 | 0.00198 | 15.90 |  | 0.2613 |
| 4A6 | 0.01289 | 0.001981 | 36.94 | * | 0.0729 | **4A6** | 0.01406 | 0.001946 | 4.46 |  | 0.7486 |
| 4D6 | 0.01318 | 0.001981 | 40.02 | * | 0.0523 | **4D6** | 0.01274 | 0.001946 | -5.35 |  | 0.6959 |
| 4E12A | 0.01326 | 0.002122 | 40.87 | * | 0.0663 | **4E12A** | 0.01594 | 0.002095 | 18.42 |  | 0.2222 |
| 4D4 | 0.01341 | 0.001981 | 42.46 | ** | 0.0395 | **4D4** | 0.01517 | 0.001946 | 12.70 |  | 0.359 |
| 4B12 | 0.01379 | 0.001981 | 46.50 | * | 0.0246 | **4B12** | 0.01408 | 0.001946 | 4.61 |  | 0.7384 |
| 4F6 | 0.01465 | 0.002015 | 55.64 | *** | 0.0084 | **4F6** | 0.01419 | 0.00198 | 5.42 |  | 0.7014 |
| 4A1B | 0.01509 | 0.002015 | 60.31 | ** | 0.0044 | **4A1B** | 0.01604 | 0.00198 | 19.17 |  | 0.1749 |
| 4F1 | 0.01579 | 0.001981 | 67.75 | *** | 0.0012 | **4F1** | 0.01377 | 0.001946 | 2.30 |  | 0.8706 |
| 4B6B | 0.01674 | 0.002015 | 77.84 | *** | 0.0003 | **4B6B** | 0.01551 | 0.00198 | 15.23 |  | 0.2816 |

**Supplementary Table 2. Root and Shoot Mean Values Trial 2**

|  |  | Strain Least Square Means Trial 2 | | | | | | |  |  |  |
| --- | --- | --- | --- | --- | --- | --- | --- | --- | --- | --- | --- |
|  | **Root Biomass** |  |  |  |  |  | **Shoot Biomass** |  |  |  |  |
| Strain | **Estimate** | **SE** | **%Increase vs Control** |  | **P Value vs Control** | **Strain** | **Estimate** | **SE** | **%Increase vs Control** |  | **P Value vs Control** |
| Control | 0.00917 | 0.000559 |  |  |  | **Control** | 0.01334 | 0.000612 |  |  |  |
| 4B6A | 0.007239 | 0.001628 | -21.06 |  | 0.2513 | **4B6A** | 0.01588 | 0.001789 | 19.04 |  | 0.1709 |
| 4B4 | 0.007551 | 0.001628 | -17.66 |  | 0.3357 | **4B4** | 0.01388 | 0.001789 | 4.05 |  | 0.7734 |
| 4A1A | 0.008527 | 0.001612 | -7.01 |  | 0.6987 | **4A1A** | 0.01377 | 0.001773 | 3.22 |  | 0.8184 |
| 4A6 | 0.008675 | 0.001628 | -5.40 |  | 0.768 | **4A6** | 0.01576 | 0.001789 | 18.14 |  | 0.193 |
| 4A3A | 0.008724 | 0.001628 | -4.86 |  | 0.7903 | **4A3A** | 0.01539 | 0.001789 | 15.37 |  | 0.2694 |
| 4E6 | 0.008748 | 0.001628 | -4.60 |  | 0.8013 | **4E6** | 0.01374 | 0.001789 | 3.00 |  | 0.8303 |
| 4A1B | 0.008884 | 0.001628 | -3.12 |  | 0.8649 | **4A1B** | 0.01392 | 0.001789 | 4.35 |  | 0.7553 |
| 4A11A | 0.008885 | 0.001156 | -3.11 |  | 0.816 | **4A11A** | 0.01428 | 0.001269 | 7.05 |  | 0.4865 |
| 4B9A | 0.009144 | 0.001612 | -0.28 |  | 0.9877 | **4B9A** | 0.012 | 0.001773 | -10.04 |  | 0.4639 |
| 4B6B | 0.009217 | 0.001612 | 0.51 |  | 0.9773 | **4B6B** | 0.0141 | 0.001773 | 5.70 |  | 0.6809 |
| 4B2 | 0.009241 | 0.001612 | 0.77 |  | 0.9661 | **4B2** | 0.01426 | 0.001773 | 6.90 |  | 0.6178 |
| 4D9 | 0.009805 | 0.001628 | 6.92 |  | 0.7055 | **4D9** | 0.01355 | 0.001789 | 1.57 |  | 0.913 |
| 4F8 | 0.009809 | 0.001628 | 6.97 |  | 0.7034 | **4F8** | 0.01523 | 0.001789 | 14.17 |  | 0.3097 |
| 4F3 | 0.009856 | 0.001612 | 7.48 |  | 0.6798 | **4F3** | 0.01443 | 0.001773 | 8.17 |  | 0.5553 |
| 4B12 | 0.01011 | 0.001628 | 10.25 |  | 0.5744 | **4B12** | 0.01455 | 0.001789 | 9.07 |  | 0.5152 |
| 4D5 | 0.01015 | 0.001628 | 10.69 |  | 0.56 | **4D5** | 0.01348 | 0.001789 | 1.05 |  | 0.9403 |
| 4A11B | 0.01027 | 0.001612 | 12.00 |  | 0.5082 | **4A11B** | 0.01243 | 0.001773 | -6.82 |  | 0.6164 |
| 4A8A | 0.01053 | 0.001612 | 14.83 |  | 0.4154 | **4A8A** | 0.01438 | 0.001773 | 7.80 |  | 0.5726 |
| 4H9 | 0.01068 | 0.001628 | 16.47 |  | 0.369 | **4H9** | 0.01357 | 0.001789 | 1.72 |  | 0.9037 |
| 4G8 | 0.01085 | 0.001628 | 18.32 |  | 0.3186 | **4G8** | 0.0129 | 0.001789 | -3.30 |  | 0.8114 |
| 4A7A | 0.01115 | 0.001628 | 21.59 |  | 0.2397 | **4A7A** | 0.01666 | 0.001789 | 24.89 | * | 0.0744 |
| 4B11A | 0.01128 | 0.001628 | 23.01 |  | 0.21 | **4B11A** | 0.014 | 0.001789 | 4.95 |  | 0.7228 |
| 4G11 | 0.01129 | 0.001628 | 23.12 |  | 0.209 | **4G11** | 0.01792 | 0.001789 | 34.33 | ** | 0.0145 |
| 4B5C | 0.01137 | 0.001628 | 23.99 |  | 0.191 | **4B5C** | 0.01661 | 0.001789 | 24.51 | * | 0.079 |
| 4A12 | 0.01141 | 0.001612 | 24.43 |  | 0.1795 | **4A12** | 0.01826 | 0.001773 | 36.88 | *** | 0.0082 |
| 4F6 | 0.01151 | 0.001628 | 25.52 |  | 0.1645 | **4F6** | 0.01248 | 0.001789 | -6.45 |  | 0.6399 |
| 4B7A | 0.01155 | 0.001628 | 25.95 |  | 0.1571 | **4B7A** | 0.01335 | 0.001789 | 0.07 |  | 0.998 |
| 4E9 | 0.01157 | 0.001628 | 26.17 |  | 0.1551 | **4E9** | 0.01483 | 0.001789 | 11.17 |  | 0.4215 |
| 4D4 | 0.01173 | 0.001628 | 27.92 |  | 0.1287 | **4D4** | 0.01827 | 0.001789 | 36.96 | *** | 0.0086 |
| 4F11A | 0.01187 | 0.001628 | 29.44 |  | 0.1099 | **4F11A** | 0.01601 | 0.001789 | 20.01 |  | 0.1509 |
| 4H1 | 0.01201 | 0.001628 | 30.97 | * | 0.0923 | **4H1** | 0.0138 | 0.001789 | 3.45 |  | 0.8071 |
| 4E12A | 0.01209 | 0.001628 | 31.84 | * | 0.0838 | **4E12A** | 0.01198 | 0.001789 | -10.19 |  | 0.4605 |
| 4F12 | 0.01217 | 0.001628 | 32.72 | * | 0.0759 | **4F12** | 0.01239 | 0.001789 | -7.12 |  | 0.607 |
| 4G6A | 0.01218 | 0.001628 | 32.82 | * | 0.075 | **4G6A** | 0.01253 | 0.001789 | -6.07 |  | 0.6582 |
| 4C6 | 0.01222 | 0.001628 | 33.26 | * | 0.0712 | **4C6** | 0.01432 | 0.001789 | 7.35 |  | 0.5965 |
| 4E1A | 0.01238 | 0.001628 | 35.01 | * | 0.058 | **4E1A** | 0.01743 | 0.001789 | 30.66 | ** | 0.0288 |
| 4F1 | 0.0124 | 0.001628 | 35.22 | * | 0.056 | **4F1** | 0.01428 | 0.001789 | 7.05 |  | 0.6141 |
| 4D6 | 0.01241 | 0.001612 | 35.33 | * | 0.0534 | **4D6** | 0.01692 | 0.001773 | 26.84 | * | 0.0528 |
| 4B10 | 0.01242 | 0.001628 | 35.44 | * | 0.0549 | **4B10** | 0.01686 | 0.001789 | 26.39 |  | 0.0589 |
| 4G12 | 0.01261 | 0.001628 | 37.51 | ** | 0.042 | **4G12** | 0.01786 | 0.001789 | 33.88 | ** | 0.0158 |
| 4G3A | 0.01267 | 0.001628 | 38.17 | ** | 0.0388 | **4G3A** | 0.01618 | 0.001789 | 21.29 |  | 0.1268 |
| 4B11B | 0.01276 | 0.001628 | 39.15 | ** | 0.0342 | **4B11B** | 0.01234 | 0.001789 | -7.50 |  | 0.5883 |
| 4F5A | 0.01277 | 0.001628 | 39.26 | ** | 0.0335 | **4F5A** | 0.01906 | 0.001789 | 42.88 | *** | 0.0024 |
| 4B7B | 0.01293 | 0.001628 | 41.00 | ** | 0.0266 | **4B7B** | 0.0134 | 0.001789 | 0.45 |  | 0.9751 |
| 4C5 | 0.01315 | 0.001628 | 43.40 | ** | 0.0189 | **4C5** | 0.01296 | 0.001789 | -2.85 |  | 0.8339 |
| 4E2 | 0.0134 | 0.001628 | 46.13 | ** | 0.0128 | **4E2** | 0.01634 | 0.001789 | 22.49 |  | 0.1065 |
| 4D1 | 0.01403 | 0.001628 | 53.00 | *** | 0.0044 | **4D1** | 0.01417 | 0.001789 | 6.22 |  | 0.6534 |
| 4G2 | 0.01461 | 0.001628 | 59.32 | *** | 0.0015 | **4G2** | 0.01632 | 0.001789 | 22.34 |  | 0.1094 |
| 4B5A | 0.01507 | 0.001628 | 64.34 | *** | 0.0006 | **4B5A** | 0.01739 | 0.001789 | 30.36 | ** | 0.0301 |
| 4B9B | 0.01624 | 0.001628 | 77.10 | *** | <.0001 | **4B9B** | 0.01451 | 0.001789 | 8.77 |  | 0.5293 |

**Supplementary Table 3. Root and Shoot Mean Values Trial 3**

|  | Strain Least Square Means Trial 3 | | | | | | | |  |  |  |
| --- | --- | --- | --- | --- | --- | --- | --- | --- | --- | --- | --- |
| Root Biomass |  |  |  |  |  | **Shoot Biomass** |  |  |  |  |  |
| Strain | **Estimate** | **SE** | **%Increase vs Control** |  | **P Value vs Control** | **Strain** | **Estimate** | **SE** | **%Increase vs Control** |  | **P Value vs Control** |
| Control | 0.009335 | 0.00061 | 0.00 |  |  | **Control** | 0.01464 | 0.001032 | 0.00 |  |  |
| 4H9 | 0.009743 | 0.001207 | 4.37 |  | 0.7563 | **4H9** | 0.01363 | 0.001768 | -6.90 |  | 0.5595 |
| 4D1 | 0.0101 | 0.001207 | 8.19 |  | 0.5632 | **4D1** | 0.01582 | 0.001768 | 8.06 |  | 0.4967 |
| 4A12 | 0.0109 | 0.001207 | 16.76 |  | 0.2383 | **4A12** | 0.01456 | 0.001768 | -0.55 |  | 0.962 |
| 4G11 | 0.01114 | 0.001207 | 19.34 |  | 0.1749 | **4G11** | 0.01509 | 0.001768 | 3.07 |  | 0.7974 |
| 4B9B | 0.01181 | 0.001207 | 26.51 | * | 0.0671 | **4B9B** | 0.0133 | 0.001768 | -9.15 |  | 0.4388 |
| 4E2 | 0.01201 | 0.001207 | 28.66 | ** | 0.0483 | **4E2** | 0.01602 | 0.001768 | 9.43 |  | 0.427 |
| 4E6 | 0.01202 | 0.001207 | 28.76 | ** | 0.0475 | **4E6** | 0.01683 | 0.001768 | 14.96 |  | 0.2106 |
| 4B5A | 0.01211 | 0.001207 | 29.73 | ** | 0.041 | **4B5A** | 0.01225 | 0.001768 | -16.33 |  | 0.1711 |
| 4B7A | 0.01239 | 0.001207 | 32.73 | ** | 0.0256 | **4B7A** | 0.01537 | 0.001768 | 4.99 |  | 0.6732 |
| 4C6 | 0.01285 | 0.001214 | 37.65 | ** | 0.0129 | **4C6** | 0.01681 | 0.001791 | 14.82 |  | 0.2246 |
| 4F1 | 0.01417 | 0.001207 | 51.79 | *** | 0.0008 | **4F1** | 0.01726 | 0.001768 | 17.90 |  | 0.1356 |

**Supplementary Table 4. Nitrogen Fixing Activity of Culturable Parviglumis Seed Endophytes**

| **Host** | **Endophyte** | **Genus** | **ARA** | ***nifH*** | **GlnLux** | **Burk's N-Free Liquid Growth** | **NH4 Secretion Trial 1** | **NH4 Secretion Trial 2** |
| --- | --- | --- | --- | --- | --- | --- | --- | --- |
| *Zea mays ssp. parviglumis* | 3G4 | Pantoea | + | + | + | Weak | Weak | Weak |
|  | 3G5 | Luteibacter | ND | ND | + | - | Weak | Moderate |
|  | 3G6 | Stenotrophomonas | - | + | + | - | Weak | Moderate |
|  | 3G7 | Pantoea | + | + | + | Weak | Weak | Weak |
|  | 3G8 | Klebsiella* | + | + | + | Strong | Strong | Strong |
|  | 3G10 | Klebsiella | + | + | + | Moderate | Weak | Moderate |
|  | 3G11 | Enterobacter | - | + | + | - | Weak | Moderate |
|  | 3A5 | Pantoea | + | + | + | - | Weak | Weak |

WGS has shown this to be *Klebsiella pneumoniae-variicola*


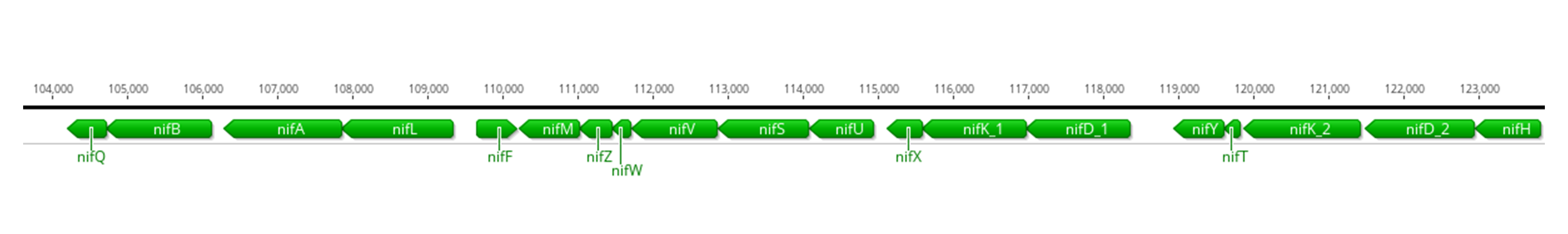


**Supplementary Figure 1.** Nitrogen fixation gene cluster from *Klebsiella pneumoniae-variicola* 4F1 whole genome sequence. Gene cluster was visualized using Geneious Prime 2021.1.1 ([https://www.geneious.com](http://www.geneious.com/))
